# *SOD2* gene polymorphisms in neovascular age-related macular degeneration and polypoidal choroidal vasculopathy

**Published:** 2009-09-09

**Authors:** Naoshi Kondo, Hiroaki Bessho, Shigeru Honda, Akira Negi

**Affiliations:** Department of Surgery, Division of Ophthalmology, Kobe University Graduate School of Medicine, Kobe, Japan

## Abstract

**Purpose:**

A nonsynonymous coding variant in the manganese superoxide dismutase (*SOD2*) gene (V16A, rs4880) has been implicated in neovascular age-related macular degeneration (AMD). However, the findings have been inconsistent. Two studies in Japanese populations reported an opposite direction of association of the same allele at the V16A variant, whereas one study in a Northern Irish population found no effect of the variant on the risk of developing neovascular AMD. To address these apparently contradictory reports, we validated the association in a Japanese population.

**Methods:**

In a Japanese population, we genotyped the V16A variant in 116 neovascular AMD patients, 140 polypoidal choroidal vasculopathy (PCV) patients, and 189 control participants. This association was also tested in a population of PCV participants to avoid variable findings across studies due to underlying sample heterogeneity and because disease phenotype was not well described in previous studies. We analyzed a tagging single nucleotide polymorphism (SNP) in addition to the V16A variant to capture all common *SOD2* variations verified by the HapMap project. Genotyping was conducted using TaqMan technology. Associations were tested using single-SNP and haplotype analyses as well as a meta-analysis of the published literature. Population stratification was also evaluated in our study population.

**Results:**

We found no detectable association of the V16A variant or any other common *SOD2* variation with either neovascular AMD or PCV, as demonstrated by both single-SNP and haplotype analyses. Population structure analyses precluded stratification artifacts in our study cohort. A meta-analysis of the association between the V16A variant and neovascular AMD also failed to detect a significant association.

**Conclusions:**

We found no evidence to support the role of any common *SOD2* variations including the V16A variant in the susceptibility to neovascular AMD or PCV. Our study highlights the importance and difficulty in replicating genetic association studies of complex human diseases.

## Introduction

Age-related macular degeneration (AMD) is a leading cause of blindness among elderly in developed nations [1]. AMD is a phenotypically heterogeneous disorder manifested at an early stage by large drusen and pigmentary abnormalities in retinal pigment epithelium (RPE). It progresses to an advanced stage by atrophy of the RPE and photoreceptors of the macula (geographic atrophy or dry AMD), or by the development of choroidal neovascularization (CNV) underneath the retina (neovascular or wet AMD) [2].

Polypoidal choroidal vasculopathy (PCV) is a hemorrhagic and exudative macular disorder that is characterized by the development of vascular networks with terminal polypoidal lesions within the inner choroid [3]. PCV is proposed to be a specific type of CNV [3,4] and much debate exists as to whether they represent different entities with distinct etiology or neovascular subsets within a common etiologic pathway [5-7]. PCV occurs much more frequently in Asians than in Caucasians, accounting for 54.7% of patients with findings suggestive of neovascular AMD in the Japanese population [8], for 24.5% in the Chinese population [9], but for only about 10% in Caucasians [3].

Numerous studies have presented evidence of a strong underlying genetic liability in AMD [10,11]. A total of four AMD risk loci have been identified with convincing statistical evidence, including the complement factor H gene (*CFH*) on chromosome 1q32 [12-17], the *ARMS2*/*HTRA1* locus on 10q26 [18-23], the complement component 3 gene on 19p13 [24-28], and two neighboring genes on 6p21: complement factor B, and complement component 2 [29-32]. These four loci are associated with both types of advanced AMD: geographic atrophy and neovascular AMD [16,22,25,29].

A large number of additional candidate susceptibility genes have been studied, but findings from most studies are inconclusive because of a lack of consistent replication [10,11]. Kimura et al. [33] reported that a nonsynonymous coding variant in the manganese superoxide dismutase (*SOD2*) gene (V16A, rs4880) was significantly associated with neovascular AMD in a Japanese population. However, a subsequent study by Esfandiary et al. [34] on a Northern Irish population reported no effect of the V16A variant on the risk of developing neovascular AMD. A recent Japanese study by Gotoh et al. [35] was also unable to replicate the initially reported association; on the contrary, this group found a significant negative association of the same allele with neovascular AMD— I.E., this variant allele was significantly protective against neovascular AMD.

To address these apparently contradictory reports, the current study evaluated the association between the V16A variant and neovascular AMD in a Japanese population. We analyzed a tagging single nucleotide polymorphism (SNP) in addition to the V16A variant to capture all common *SOD2* variations verified by the HapMap project [36]. Therefore, there was an increased coverage of this gene in our study as compared to the two previous studies in Japanese populations, which only examined the V16A variant [33,35]. We also tested for their association with PCV because the disease phenotype was not well described in previous studies [33,35]. Particularly, the initial study by the Japanese group did not consider the findings from indocyanine green (ICG) angiography in their evaluation [33], which is the only way to obtain a clear image of PCV lesions. This raises the possibility that their cohort may have included a measurable amount of PCV given its high prevalence in the Japanese population [8]. It has been suggested that attention to phenotype classification is a key aspect of genetic studies of AMD, to avoid variable findings across studies due to underlying sample heterogeneity [37]. Additionally, we performed a meta-analysis to assess the overall effect of the V16A variant on neovascular AMD across the different independent studies.

## Methods

### Study participants

This study was approved by the Institutional Review Board at Kobe University Graduate School of Medicine and was conducted in accordance with the Declaration of Helsinki. Written informed consent was obtained from all participants. All case and control participants enrolled in this study were Japanese individuals recruited from the Department of Ophthalmology at Kobe University Hospital in Kobe, Japan. The majority of participants had participated in our previous studies [38,39] in which phenotyping criteria were fully described. In brief, all our neovascular AMD and PCV patients underwent comprehensive ophthalmic examinations including ICG angiography, and were defined as having angiographically well defined lesions of CNV or PCV. The control participants, who were not related to the case participants, were defined as individuals without macular degeneration and changes such as drusen or pigment abnormalities, and thus were categorized as having clinical age-related maculopathy staging system stage 1 [40]. The demographic details of our study population are presented in Table 1.

**Table 1:** Characteristics of the study population.

**Groups**	**Neovascular AMD**	**PCV**	**Controls**
Number of subjects	116	140	189
Gender (male/female)	91/25	108/32	114/75
Mean age±SD (years)	75±7.2	73±6.9	72±5.8
Age range (years)	57–91	57–86	56–95

A total of 116 subjects with neovascular AMD, 140 with PCV, and 189 control participants were enrolled in the present study. The gender breakdown, mean age, and age range of each population are shown. Abbreviations: age-related macular degeneration (AMD); polypoidal choroidal vasculopathy (PCV); standard deviation (SD).

### Marker selection

To comprehensively and effectively cover common variations in the *SOD2* locus, we ran the Tagger tool [41] from the HapMap project database [36] for the Japanese in Tokyo (JPT) population. The minor allele frequency (MAF) cutoff was set at 0.05; the r^2^ cutoff was set at 0.9; and the Tagger Pairwise mode was used. Two SNPs, the V16A variant (rs4880) and rs5746136, were selected for genotyping. On the basis of the HapMap JPT data, these two SNPs captured all seven HapMap SNPs in *SOD2*, with a MAF greater than 5% and a mean r^2^ value of 1.0. Therefore, this set of two SNPs is representative of the common genetic variations in *SOD2* because it acts as a proxy marker for other untyped SNPs in this locus.

### SNP genotyping

Genomic DNA was extracted from peripheral blood immediately after it was drawn using QIAamp DNA Blood Maxi Kit (Qiagen, Valencia, CA) according to the manufacturer’s instructions. Genotyping was conducted using TaqMan^®^ SNP Genotyping Assays (Applied Biosystems, Foster City, CA) on a StepOnePlus™ Real-Time PCR system (Applied Biosystems), in accordance with the manufacturer’s instructions.

### Statistical analysis

Testing for association was performed using a software package PLINK v1.00 [42]. Deviations from Hardy–Weinberg equilibrium were tested using the exact test [43] implemented in PLINK. The two SNPs reported in the present study did not show significant deviation from Hardy–Weinberg equilibrium in neovascular AMD, PCV, or control participants (all p>0.05). Single-marker association analyses were performed using the χ^2^-test or Fisher’s exact test under allele (1 degree of freedom, df), genotypic (2 df), dominant (1 df), and recessive (1 df) genetic models. To adjust for differences in age and sex between case and control subjects, we performed logistic regression analyses using SNPStats software [44], assuming an additive genetic model by fitting age and sex as continuous and categorical covariates, respectively. A p>0.05 was considered statistically significant. Measures of linkage disequilibrium (LD) and haplotype association statistics were calculated using Haploview software [45]. Omnibus tests of haplotype associations were performed with PLINK.

Power calculations were conducted using QUANTO version 1.2 [46]. Assuming an additive genetic model, we had 80% power to detect an association of the V16A variant with an odds ratio (OR) ≥1.79 (or ≤0.47) for the neovascular AMD sample, and ≥1.70 (or ≤0.51) for the PCV sample.

Hidden population stratification in genetic association studies can generate a spurious positive or negative association [47]. To prevent potential stratification in our study cohort, population stratification was evaluated using STRUCTURE software [48] as described in previous studies [38,39,49,50]. The following 38 polymorphic SNPs, which are not in LD with each other (r^2^<0.04), were used for this analysis: rs3818729 (1p13.2), rs696619 (1p21.3), rs9434 (1p36.12), rs1554286 (1q32.1), rs13388696 (2p23.1), rs1042034 (2p24.1), rs10932613 (2q35), rs7641926 (3p26.2), rs2305619 (3q25.32), rs4074 (4q13.3), rs6876885 (5p15.1), rs6459193 (6p11.2), rs3779109 (7p22.1), rs2227667 (7q22.1), rs6468284 (8p12), rs10757278 (9p21.3), rs955220 (9p24.3), rs1927911 (9q33.1), rs4838590 (10q11.22), rs12806 (10q24.2), rs2019938 (11p15.5), rs609017 (11q24.3), rs3912640 (12p13.2), rs2283299 (12p13.33), rs715948 (12q13.3), rs7328193 (13q12.11), rs1048990 (14q13.2), rs911669 (14q32.13), rs16948719 (15q22.31), rs11076720 (16q24.3), rs1051009 (17p13.2), rs1292033 (17q23.1), rs7239116 (18q11.2), rs892115 (19p13.2), rs3826945 (19p13.3), rs844906 (20p11.21), rs2825761 (21q21.1), and rs3884935 (22q13.1). The log likelihood of each analysis with a varying number of populations (*K*) was computed from three independent runs (20,000 burn-in and 30,000 iterations). The best estimate of *K* was defined by calculating posterior probabilities (*Pr*, *K*=1, 2, 3, 4, or 5) based on the log likelihood, as described by Pritchard et al. [51].

A meta-analysis was performed using R and StatsDirect software (StatsDirect, Cheshire, UK). Data from our own study and two earlier case-control studies performed by Kimura et al. [33] and Gotoh et al. [35] were used for the meta-analysis. The study by Esfandiary et al. [34] was not included in the meta-analysis, because the allele and genotype data were unavailable. A summary OR was calculated using the random-effects model of DerSimonian and Laird [52]. Heterogeneity between studies was tested using Cochran’s Q statistic [53,54] and the *I*^2^ statistic for inconsistency [53,54]. *I*^2^ is a measure of the proportion of total variation across studies due to heterogeneity beyond chance. *I*^2^ is provided by the formula *I*^2^=100% × *Q* − (*k* − 1)/*Q*, where *Q* is the Cochran’s heterogeneity statistic and *k* is the number of studies. The *Q*-test is known to have poor power if there are few studies and is typically considered statistically significant at p<0.1 [53,54]. On the other hand, *I*^2^ is unaffected by the number of studies, and it is regarded as large for values >50% [55].

## Results

We genotyped V16A (rs4880) to validate the previously reported association and this SNP was supplemented by an additional SNP rs5474613 to increase coverage of the variations in *SOD2*. These two SNPs captured all common *SOD2* SNPs (MAF > 0.05) observed in the HapMap JPT subjects with a mean r^2^ of 1.0. The allele and genotype counts and results of single-SNP association analysis are given in Table 2. Neither of the two SNPs showed a significant association with either neovascular AMD or PCV in any of the genetic models (all p>0.05; Table 2). Adjustment for age and sex by logistic regression analyses under an additive model did not affect this conclusion (neovascular AMD, p=0.18 and 0.76 for rs4880 and rs5474613, respectively; PCV, p=0.25 and 0.83 for rs4880 and rs5474613, respectively).

**Table 2:** Allele and genotype distributions of rs4880 (V16A) and rs5746136 and the results of association tests

**SNP**	**Status**	**Genotype count (frequency)**			**Allele 2 frequency**	**OR_allele_ (95% CI)**	**p value**			
		**11**	**12**	**22**			**Allele (1 df)**	**Genotype (2 df)**	**Dominant (1 df)**	**Recessive (1 df)**
rs4880	Control	137 (0.725)	44 (0.233)	8 (0.042)	0.159	-	-	-	-	-
	AMD	90 (0.776)	24 (0.207)	2 (0.017)	0.121	0.73 (0.45–1.18)	0.19	0.44	0.35	0.33
	PCV	105 (0.750)	33 (0.236)	2 (0.014)	0.132	0.81 (0.52–1.26)	0.34	0.38	0.7	0.2
rs5746136	Control	77 (0.407)	85 (0.450)	27 (0.143)	0.368	-	-	-	-	-
	Neovascular AMD	49 (0.422)	53 (0.457)	14 (0.121)	0.349	0.92 (0.66–0.30)	0.64	0.86	0.8	0.58
	PCV	57 (0.407)	61 (0.436)	22 (0.157)	0.375	1.03 (0.75–1.42)	0.85	0.93	1	0.72

Neither of the two SNPs showed a significant association with either neovascular AMD or PCV in any of the genetic models. Alleles 1 and 2 represent major and minor alleles, respectively, and 11, 12, and 22 represent the homozygote of allele 1, heterozygote, and homozygote of allele 2, respectively. The *T* and C alleles of rs4880 are labeled as alleles 1 and 2, respectively. The *G* and *A* alleles of rs5746136 are labeled as alleles 1 and 2, respectively. The genotypic, dominant, and recessive tests for rs4880 were performed using the Fisher’s exact test, and the others were performed by the χ^2^-test. The dominant model compared a combination of heterozygotes and rare homozygotes to the common homozygotes. The recessive model compared the rare homozygotes to a combination of common homozygotes and heterozygotes. OR_allele_ indicates per-allele odds ratio in the allelic test. Abbreviations: single nucleotide polymorphism (SNP); age-related macular degeneration (AMD); polypoidal choroidal vasculopathy (PCV); odds ratio (OR); confidence interval (CI); degree of freedom (df).

The two SNPs genotyped were in moderate LD with each other (D´=0.75) and haplotype association analyses were conducted using these two SNPs. No significant haplotype associations were found for either neovascular AMD (omnibus p=0.51, 3 df; Table 3) or PCV (omnibus p=0.80, 3 df; Table 3).

**Table 3:** Results of a haplotype-based association study

Haplotype*	**Control frequency**	**Neovascular AMD**			**PCV**		
		**Frequency**	**p value**	**OR (95% CI)**	**Frequency**	**p value**	**OR (95% CI)**
TG	0.489	0.540	0.23	1.22 (0.88–1.70)	0.504	0.72	1.06 (0.78–1.44)
TA	0.352	0.339	0.75	0.95 (0.67–1.33)	0.364	0.74	1.06 (0.76–1.46)
CG	0.143	0.110	0.26	0.75 (0.45–1.23)	0.121	0.42	0.83 (0.52–1.31)
CA	0.016	0.010	0.53	0.63 (0.14–2.81)	0.011	0.57	0.67 (0.17–2.71)

*The alleles of single nucleotide polymorphisms forming haplotypes are presented (from left to right, rs4880 and rs5746136). The *P* values were calculated by the χ^2^-test on haplotype counts (1 degree of freedom [df]). Omnibus haplotype tests based on the four haplotypes were not significant (omnibus p=0.51, 3 df for neovascular AMD; omnibus p=0.80, 3 df for PCV). Abbreviations: age-related macular degeneration (AMD); polypoidal choroidal vasculopathy (PCV); odds ratio (OR); confidence interval (CI).

Next, population stratification was evaluated by STRUCTURE [48] using 38 unlinked genome-wide SNPs. No evidence of stratification was found in our study cohort [*Pr* (*K*=1>0.99)] indicating that population stratification did not account for the results observed in the present study.

**Figure 1:**
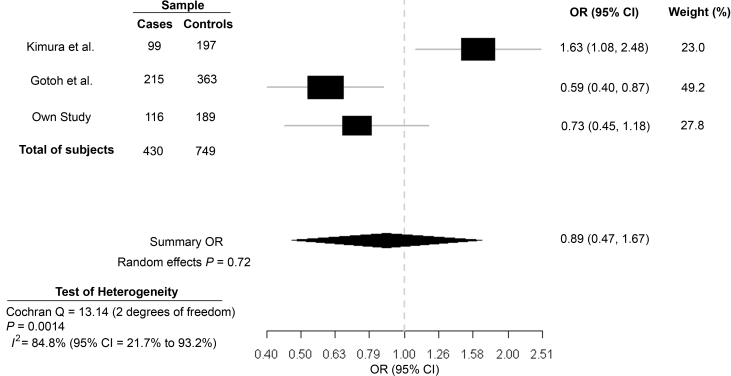
Meta-analysis of the V16A variant for its association with neovascular AMD. Odds ratios (ORs, black squares) and 95% confidence intervals (CIs, bars) are presented for each study. Also shown is the shaded diamond of the summary OR using the random-effects model of DerSimonian and Laird [52]. The genotype data included in the meta-analysis refer to the description of Kimura et al. [33] and Gotoh et al. [35]. Heterogeneity between studies was tested using Cochran’s Q statistic [53,54] and the *I*^2^ statistic for inconsistency [53,54]. Abbreviations: AMD represents age-related macular degeneration.

Finally, a meta-analysis was performed to assess the association of the V16A variant with neovascular AMD across the different independent studies. Association results from our study (only neovascular AMD) and the two previous Japanese studies [33,35] were combined using the random-effects model of DerSimonian and Laird [52], and a summary OR for the model was calculated based on the allele frequency data. As shown in Figure 1, the heterogeneity test across studies (Cochran’s *Q* statistic) was significant for this variant (p=0.0014), and inconsistency of the genetic effects across the three studies was high (*I*^2^=84.8%). No significant association was detected for the V16A variant, with a random-effects summary OR of 0.89 (95% CI, 0.47–1.67). Allele and genotype frequencies of the V16A variant observed in the two previous Japanese studies [33,35] are shown in Table 4.

**Table 4:** Allelic distributions of the V16A variant reported by earlier studies

**Study group**	**Kimura et al.**		**Gotoh et al.**	
Subjects	Case	Control	Case	Control
Number of subjects	99	197	215	363
Genotype (%)				
TT	59.6	67.5	81.9	73.8
TC	31.3	31.5	18.1	23.4
CC	9.1	1.0	0	2.8
Minor allele frequency (%)	24.7	16.8	9.1	14.5
Allelic P-value	0.020	-	0.0073	-

The genotype data in Table 3 refer to descriptions made by Kimura et al. [37] and Gotoh et al. [35]. Minor allele frequency of the V16A variant in case subjects were widely divergent; much higher frequency of the variant allele was observed in the Kimura et al.’s study.

## Discussion

To validate the previously described associations of the *SOD2* V16A variant with neovascular AMD in Japanese populations [33,35], we examined this variant together with an additional SNP to increase coverage of the gene in an independent Japanese population with neovascular AMD. These two SNPs are perfect surrogates of all *SOD2* SNPs identified by the HapMap project [36], and are representative of the common *SOD2* variations. We also tested for an association with PCV, given the possibility that the study cohort in previous Japanese studies might have had a measurable amount of PCV [33,35]. We found no detectable association of the V16A variant or any other common *SOD2* variation with either neovascular AMD or PCV by single-SNP or haplotype analysis. Population structure analyses precluded stratification artifact in our study cohort. A meta-analysis of the association between the V16A variant and neovascular AMD also failed to detect a significant association.

SOD2 plays a crucial role in the detoxification of superoxide free radicals, which protects cells from reactive oxygen species–induced oxidative damage [56]. Oxidative stress is a hypothesized pathway for the pathophysiology of AMD [56], and *SOD2* is a reasonable candidate gene for the disease. Kimura et al. [33] and Gotoh et al. [35] reported that opposite alleles at the same variant V16A in the *SOD2* gene are positively associated with neovascular AMD in Japanese populations. This phenomenon, referred to as “flip-flop” associations, may serve as additional evidence of a true genetic association in populations of different ancestry (i.e., when noncausal variants are tested, observed effects of the variants can vary between studies because of differences in their correlation with other causative variants) [57]. Theoretically, flip-flop associations for a genuine causative variant would not occur in samples of the same ethnic origin and are often regarded as spurious findings [57].

Minor allele frequencies of the V16A variant in control subjects were similar among the two earlier Japanese studies and our own study: 16.8% in the studies by Kimura et al. [33], 14.5% in Gotoh et al. [35], and 15.9% in the present study. However, minor allele frequencies of the V16A variant in case subjects were widely divergent; 24.7% in the studies by Kimura et al. [33], 9.1% in Gotoh et al. [35], and 12.1% in the present study. This inconsistency could possibly be due to differences in case selection criteria. Given the possibility that the original study might include a measurable number of PCV subjects, the potential role of the V16A variant in PCV was also explored in the present study. We found that the allele and genotype distributions of this variant were very similar between subjects with PCV and neovascular AMD, and the association results were consistent across these two phenotypes. Another possible reason for the much higher frequency of the V16A variant allele observed in the initial study is genotyping error. There are between-study differences in genotyping methods. The initial study used polymerase chain reaction restriction fragment length polymorphism analysis to genotype this variant [33], whereas we and Gotoh et al. [35] employed the TaqMan technology, which is now a well established technology for genotyping the V16A variant [58,59]. As shown in previous studies [60,61], different genotyping technologies can yield inconsistent genotyping results.

*SOD2* maps to chromosome 6q25.3, a region that has not been implicated in AMD by any genome-wide scans [10]. To further validate its association, we accessed the NEI/NCBI dbGAP database, which provides results of genome-wide association study on 395 individuals with AMD and 198 controls from the National Eye Institute Age-Related Eye Disease Study (AREDS). This study looked at three *SOD2* SNPs, including the V16A variant and two other SNPs (rs8031 and rs2855116), and found no significant association (all nominal p>0.1), confirming our findings and those of Esfandiary et al. [34].

In conclusion, we found no evidence to support the role of any common *SOD2* variation including the V16A variant in the susceptibility to neovascular AMD or PCV. Our study highlights the importance and difficulty in replicating genetic association studies of complex human diseases. The only way to have complete confidence in genetic association is by conducting independent replications. Further replications will allow a definitive conclusion regarding the etiological relevance of this variant in AMD.
